# Recent advances in understanding the roles of the enteric nervous system

**DOI:** 10.12703/r/11-7

**Published:** 2022-03-24

**Authors:** Atchariya Chanpong, Osvaldo Borrelli, Nikhil Thapar

**Affiliations:** 1 Neurogastroenterology & Motility Unit, Gastroenterology Department, Great Ormond Street Hospital for Children, London, WC1N 3JH, UK; 2 Department of Pediatrics, Faculty of Medicine, Prince of Songkla University, Songkhla, 90110 Thailand; 3 Stem Cells and Regenerative Medicine, UCL Great Ormond Street Institute of Child Health, London, WC1N 1EH, UK; 4 Gastroenterology, Hepatology and Liver Transplant, Queensland Children’s Hospital, Brisbane, Queensland 4101, Australia; 5 School of Medicine, University of Queensland, Brisbane, Australia; 6 Woolworths Centre for Child Nutrition Research, Queensland University of Technology, Brisbane, Australia

**Keywords:** Enteric Nervous System, enteric neurons, enteric glia, microbiota-gut-brain axis, early life programming, enteric neuronal plasticity, GI motility disorders

## Abstract

The enteric nervous system (ENS), the intrinsic innervation of the gastrointestinal (GI) tract, is a vast, mesh-like network of neurons and glia embedded within the bowel wall. Through its complex circuitry and neuronal diversity, the ENS is capable of functioning autonomously but is modulated by inputs from the central nervous system (CNS). The communication between the ENS and CNS is bidirectional and, together with crosstalk of these systems with microbiota housed within the GI tract, underpins the so-called microbiota-gut-brain axis. The ENS functions as a master regulator and coordinates many of the essential functions of the body, including GI motility, sensation and secretion. It is also capable of interacting with other cells, including intestinal epithelial, neuroendocrine and immune cells, to regulate their development as well as structural and functional integrity. Disruption of these ENS interactions, especially during early life, is likely to contribute to the aetiopathogenesis of disorders of the GI tract as well as elsewhere in the body, including neurodegenerative diseases. In this article, we highlight recent advances in our understanding of the roles of the ENS, especially in its complex and reciprocal interactions that influence GI motility, sensation, intestinal epithelial integrity, immunity and neuroendocrine function, particularly focusing on the influence of the ENS in early life and early life programming.

## Introduction

The enigmatic enteric nervous system (ENS), the intrinsic innervation of the gastrointestinal (GI) tract, is a vast, mesh-like network of neurons and glia embedded within the bowel wall. It is the largest subdivision of the peripheral nervous system, receiving inputs from both the sympathetic and parasympathetic nervous systems^1^. Although the ENS is capable of functioning autonomously, it communicates with, and can be modulated by inputs from, the central nervous system (CNS). This communication between the CNS and ENS is bidirectional and underpins one of the most fascinating interactions of the human body, the so-called brain-gut axis^2,3^. A considerable body of research has additionally shown the vital interaction of gut microbiota at the ENS interface, instigating the adage ‘brain-gut-microbiome axis’^3,4^.

To its end, the ENS not only is primarily responsible for GI motility, sensation and secretion but also appears capable of interacting with a host of other cells, including intestinal epithelial, neuroendocrine and immune cells, to regulate their development as well as structural and functional integrity^1^. This breadth of roles and interactions is perhaps not surprising. It could be argued that, though generally designated as the body’s ‘second brain’^5^, the ENS, in fact, is more deserving of the title ‘first brain’ given that in the most primordial forms of life it developed before and independent of the CNS^6^. Not only does the ENS contain a massive number of neurons, nearly equal to that of the spinal cord, but it also has a neuronal diversity to match. Indeed, in simpler life forms, the ENS coordinates many of the essential and diverse functions of the body, and increasing evidence shows that it retains this role in even the most complex organisms, such as humans.

In this article, we highlight recent advances in our understanding of the roles of the ENS, especially in its complex and reciprocal interactions with a range of cell types and organ systems that ultimately influence GI motility, sensation, intestinal epithelial integrity, immunity and neuroendocrine function. Disruption of these interactions, especially during early life, is likely to contribute to the aetiopathogenesis of disorders of the GI tract and elsewhere in the body.

## ENS development and organisation

ENS development will not be covered here in any detail except to highlight recent key progress. It is comprehensively described in excellent reviews^1,7–9^.

In brief, the ENS along the entire length of the GI tract is derived from neural crest-derived progenitors, originating mostly from the vagal region of the neural tube, and the distal bowel receives an additional contribution from the sacral neural crest^10^. These precursor cells, through a number of signalling pathways and molecules, differentiate into neurons and glia and migrate along the entire length of the developing intestine^1^. The mature ENS comprises the myenteric (Auerbach) and the submucosal (Meissner) plexuses, which consist of a vast array of neural cells classified on the basis of their morphology, chemical coding and electrophysiological properties into distinct functional classes of neurons, and glia^11^. The ENS that emerges from this complex process is exquisite in its function but rather haphazard in its overt appearance. More recently, the field has focussed on the key effector cells that regulate motility as well as how functional circuits are established and precise motility generated.

## ENS neuronal plasticity and turnover

The ENS has a remarkable capacity for growth, plasticity and repair, which is probably essential for its critical role in allowing the organism to adapt to changes in the internal or external environment. This is especially important given that the ENS repeatedly faces insults from mechanical, chemical and infectious agents. Until recently, there was little evidence of the occurrence of post-natal neurogenesis despite reports of the isolation and harvesting of multipotent ENS progenitors from the post-natal GI tract, even well into adult life^12,13^. A number of studies suggested that enteric neurogenesis is absent beyond early post-natal life or, where it could be seen, emanates from enteric glia in a limited capacity in response to significant insults such as chemical injury^14–16^. Kulkarni *et al*.^17^, however, showed that the adult myenteric ganglia were capable of maintaining neuronal numbers despite evidence of ongoing neuronal loss, suggestive of a concurrent process of neuronal replenishment. The authors further established that the progenitors effecting neurogenesis were not glial given that they expressed the neuro-epithelial stem cell marker Nestin rather than Sox10. Furthermore, disruption of cell cycle regulation in Nestin^+^ cells resulted in enteric neuronal hyperplasia. Overall, these novel and rather incredible findings of massive neuronal turnover in the adult gut need to be further and robustly explored and verified. If these findings are confirmed, clarity over potential triggers for this neurogenesis will need to be elucidated^18^. The potential of significant enteric neuronal turnover perhaps most pronounced in early (including post-natal) life raises the possibility that a number of GI motility disorders arise from disruption of this process via a number of different mechanisms (e.g., dysbiosis, dietary changes, and infections). As discussed later, a number of researchers, contrary to the findings of Kulkarni *et al*., have suggested a role for glia in neurogenesis in the adult gut^15,19^.

## Disruption of the ENS in early life: early life programming

The concept of early life vulnerability and the importance of the first 1000 days (from conception to the second year of life) are highly topical and thought to be key determinants of health and disease. Indeed, development of the ENS appears to extend beyond embryogenesis and foetal life well into post-natal life as evidenced by changing patterns of motility, making it particularly vulnerable to re-programming. Thus, any disturbances in ENS development or disruptions of its complex molecular interactions can lead to structural or functional GI abnormalities^1^. Interestingly, not only can this result in immediate effects but, akin to cardiovascular programming, these early programming events can have consequences after significant periods of time, perhaps coinciding with other influences on ENS function, such as puberty. Functional abdominal pain disorders, now referred to as disorders of gut-brain interaction, appear to be good examples of this (reviewed in Thapar *et al*.^20^). A number of early life factors and ‘insults’ appear to programme the ENS and lead to disturbances of its function.

It has long been known that breast milk contains neurotrophic factors (e.g., glial cell line-derived neurotrophic factor, transforming growth factor β and ciliary neurotrophic factor), cytokines and oligosaccharides that are important for the development and neuronal survival of the ENS^21,22^. Fichter *et al*. demonstrated that dissociated myenteric neurons from post-natal rats generated longer lengths of neurite outgrowth and displayed higher neuronal survival when cultured in a medium enriched with breast milk protein extracts, in comparison with the control medium^21^. In similar experiments, other milk-derived bioactive peptides such as tryptic β-casein hydrolysate were also found to modulate ENS maturation by stimulating neurite outgrowth and forming enteric ganglia-like structures^23^.

It is well recognised that GI infections in children predispose them to develop functional abdominal pain disorders^24,25^. A number of studies show the likely contribution of inflammatory damage to neuronal hyper-excitability, possibly related to aberrant ENS regeneration^26^. Similar pathophysiology with cow’s milk protein allergy may also lead to disorders of gut-brain interaction in later life^27,28^. Recent studies have suggested that infections *in utero* may impact on ENS integrity. Though yet to be confirmed in humans, chorioamnionitis in animal models was associated with ENS damage, including loss of both neurons and glia^29^.

In germ-free mice with a complete absence of gut microbiota in early post-natal life, the ENS develops with fewer myenteric neurons but with a higher proportion of neuronal nitric oxide synthase (nNOS) subtypes^30^. Intestinal motility was also reduced in these animals, affecting both the frequency and amplitude of intestinal contractions. Likewise, neonatal mice that received antibiotics appear to have disruption of microbiota composition and diversity in the small and large intestine^31^. Early post-natal exposure of mouse pups to vancomycin (from birth to post-natal day 10) affected colonic motility, reduced the density of myenteric neurons and proportions of nNOS neurons and increased proportions of calbindin (cholinergic) neurons^31^. The impact of vancomycin on the ENS appears to vary with post-natal age and is likely secondary to influences on the microbiota, although direct effects on the ENS are possible^32,33^. Short-chain fatty acid (SCFA)-producing gut microbiota can modulate extrinsic enteric-associated neurons, composed of sensory afferents and autonomic efferents, to regulate GI motility^34^. SCFAs (i.e., acetate, propionate and butyrate) at physiologically relevant concentrations could regulate ENS development by increasing the growth rate of human neural progenitor cells through the expression of genes involved in neurogenesis, proliferation and apoptosis. Conversely, high levels of SCFAs are associated with toxic effects on the neural progenitor cells^35^. Other bacterial metabolites, including branched SCFA, isovaleric acid, also cause colonic smooth muscle relaxation^36^. These findings underpin the importance of the intestinal microbiota to the post-natal development of the ENS^30,37^. Interestingly, these effects on the ENS can be rescued early in post-natal life through replenishment of microbiota^37,38^ (reviewed in 37).

In addition to intestinal dysmotility, other studies in germ-free mice also revealed stress and anxiety-like behaviour, compared with mice with healthy gut microbiota. These changes were difficult to reverse in later life^39–41^. This highlights the fact that alongside their effects on the ENS, gut microbiota also impact upon the CNS. These complex and wide-ranging influences within the axis are mediated by the immune system and extrinsic innervation as well as a number of factors, including microbial products (e.g., SCFAs and lipopolysaccharide) and hormones^42^. Several studies in mice have confirmed that gut microbial composition can modulate the development, maturation and function of the CNS both pre- and post-natally^41,43–45^. Importantly, there is a developmental “window of vulnerability” early in life when perturbation of the gut microbiota causes long-lasting effects on CNS function. An example where disruption of the ENS and gut microbiome in humans is implicated in the development of lifelong consequences can be seen in children with autistic spectrum disorders^46–50^. Furthermore, GI dysfunction (e.g., abdominal pain, constipation, delayed gastric emptying and colonic transit and weight loss) has been noted in patients with amyotrophic lateral sclerosis, Parkinson’s disease^51–54^ or Alzheimer’s disease^55^ prior to the onset of CNS manifestations of these disorders. Data from patients as well as animal models suggest that Parkinson’s disease affects distinct subsets of neurons and glia in the ENS. Several studies have revealed Lewy-type pathology in enteric neurons from biopsies of patients with Parkinson’s disease^56,57^ and in animal models^58,59^. This type of pathology in the CNS is associated with degeneration of dopaminergic neurons; however, in the ENS, the association with neurodegeneration is less clear. A reduction in VIP expression has been described in the submucosal plexus of Parkinson’s patients with constipation^60^. Scheperjans *et al*. demonstrated differences in the abundance of certain bacterial families in faecal microbiomes between patients with Parkinson’s disease and controls^61^. There were associations between clinical parameters (e.g., postural instability and gait difficulty) and microbiota (e.g., Enterobacteriaceae)^61^. Another study found a reduction in faecal SCFA concentrations in Parkinson’s disease that theoretically could cause ENS disturbances leading to GI dysmotility^62^. However, the role of early life disruption of the ENS and microbiota in these neurodegenerative disorders is as yet undetermined (reviewed in 52,63).

Environmental factors such as physical and emotional stress at early stages of life also have potential impacts on ENS development and its lifelong physiological function. These factors lead to various GI disorders such as abdominal pain, diarrhoea, constipation and increased susceptibility to enteric infections^1,64^. Early exposure to environmental stressors has been known to influence microbial composition in the gut, mediated through the hypothalamic-pituitary-adrenal axis, via the activation of corticotropin-releasing factor^44,65^. This results in the alterations of intestinal epithelial integrity, intestinal motility and secretions, which contribute to the change of the intestinal habitat of resident bacteria, leading not only to dysbiosis but also to abnormal stress responses^44,65–67^. Other mechanisms of stress-induced GI diseases involve an increase in the number and activation of immune cell (mast cell) and GI neurotransmitters, including choline acetyltransferase (ChAT), substance P and serotonin^64^. In animal models^64^, neonatal maternal separation induces long-term upregulation of ChAT activity, leading to an increase in GI motility, mast cell degranulation and visceral hypersensitivity. The GI pathophysiological changes in animals exposed to early-life stressors are comparable to those proposed in humans with irritable bowel syndrome.

Though only explored within animal models but not proven in humans, deficiencies of certain nutritional components (e.g., vitamin A) or exposure to pharmacological agents (e.g., ibuprofen) commonly used in pregnancy appear capable of disrupting normal ENS development^68,69^. In these experiments, the ENS effects, including aganglionosis, were structurally significant, raising the possibility that more subtle ENS changes with functional consequences occur with environmental or dietary agents during both pregnancy and the early post-natal period.

## ENS and GI motility

The control of GI motility and the propagation of luminal contents is an essential function of the ENS. This is reliant on having both a repertoire of appropriate cellular components and patterns of communication between them (i.e., circuits) to initiate and regulate precise contractile events along the length of the GI tract.

Beyond excitatory and inhibitory neurons, the regulation of motility also appears to rely upon the cooperation of the ENS with other key cell types, notably platelet-derived growth factor receptor alpha (PDGFRα), interstitial cells of Cajal (ICCs), as well as enteric glial cells (EGCs) and intestinal macrophages. Although interactions of the ENS with smooth muscle, ICCs and PDGFRα^+^ cells have long been implicated in GI motility, as multicellular units referred to as SIP syncytium (reviewed in 70,71), only recently has the interaction of intestinal macrophages to modulate GI motility and shape ENS circuits been described. This, as well as the role of EGCs in motility, is discussed in the sections below.

Unfortunately, little is known regarding the generation of neuronal diversity and factors involved in the specification of individual neuronal subtypes within the ENS. Using comparative RNA profiling, Memic *et al*. showed that a lack of SOX6 in the ENS reduced the numbers of gastric dopamine neurons and resulted in delayed gastric emptying, suggesting that SOX6 is required for the development of this neuronal subtype^72^. Using single-cell RNA sequencing, a recent study revealed a novel classification of myenteric neurons, based on their communication features, and provided a better understanding of the development of neuronal diversity in the ENS^73^. Lasrado *et al*., using fate mapping of ENS precursors, showed that lineage relationships underpin the spatial and functional organisation of intestinal neural networks^74^. Founder precursors gave rise not only to a diversity of neuronal and glial subtypes but also to ENS clonal units that colonise and organise themselves spatially across the gut wall as columns along the serosa-mucosa polar axis of the gut cylinder. Importantly, the clonal units also showed coordinated activity, suggesting that overlapping clonal units underpin the structure and function of the plexuses of the ENS, which ultimately may explain the coordination of gut motility and secretions along the GI tract^74^. Sasselli *et al*.^75^ showed that ablation of planar cell polarity genes such as *Ceslr3* results in disruption of the organisation of enteric neuronal projections and presumably in circuitry with profound effects on GI motility. Akin to a significant proportion of GI motility disorders, the ENS in *Celsr3* mutants showed minimal obvious visible changes in the ENS in terms of numbers of cells or ganglia, neuronal diversity and ENS networks^75^.

## Enteric glial cells

EGCs are the most prolific components of the ENS that intimately surround enteric neurons and nerve fibres. However, beyond the traditional concept of functioning as neuronal supporting structures, they are now recognised to play key roles in regulating a range of physiological processes, many previously considered to be under the exclusive remit of enteric neuronal cells. Such processes are now known to occur through bidirectional interactions with cells, both neuronal and non-neuronal, in the gut wall^76^.

Owing to the diversity of EGCs along the GI tract, they have been classified into at least six different subtypes according to their regional localisation^76,77^. Intraganglionic EGCs reside in apposition to neuronal cell bodies within either the myenteric (myenteric glia) or submucosal (submucosal glia) plexuses, modulating the activity of myenteric and secretomotor neurons, respectively^78,79^. Myenteric glia are arguably the best studied and appear to be involved in a number of functions, including regulation of neuroinflammation and oxidative stress, gliogenesis, neurogenesis, and replenishment of mucosal glia (reviewed in 76). Extraganglionic EGCs are associated with nerve fibres at different levels and comprise enteric plexus, interganglionic, mucosal and intramuscular glia. Interganglionic glia appear to be involved in neuromodulation and signal propagation in the glial network; whereas, mucosal glia appear to influence epithelial cell maturation and modulate both immune responses and neuroendocrine signalling (reviewed in 76).

EGCs appear to play key roles in maintaining the cellular integrity of the ENS, including neurons. Though not supported by the findings by Kulkarni *et al*. (discussed earlier), Laranjeira *et al*. showed that after chemical injury to the intestine EGCs act as the source of new neurons, suggesting that in the face of enteric neuronal loss from insults and the absence of constitutive neurogenesis, EGCs may act to sustain enteric neuronal populations^15^. McCallum *et al*.^19^ recently demonstrated that non-neuronal cells in zebrafish, presumed to be EGCs, were dynamic, proliferating under physiological conditions and differentiating into enteric neurons. Kabouridis *et al*. showed that EGCs migrate from the plexuses of the gut wall to the lamina propria, where they replenish mucosal EGCs and that, importantly, this process appears to be regulated by luminal microbiota^45,80^. Indeed, the formation of the EGC network appeared to parallel the evolution of the gut microbiota, which also carries considerable potential functional implications for the microbiota-gut-brain axis.

EGCs are also considered to have essential roles in the maintenance of intestinal epithelial barrier integrity and regulation of epithelial cell proliferation^81^. Studies in animals show that EGCs have antiproliferative and prodifferentiative effects on epithelial cells^82,83^ and that their loss leads to an increase in intestinal permeability^84,85^. Mucosal glia have intricate interactions with nerves, immune cells, microbiome, enterocytes and/or enterochromaffin (neuroendocrine) cells at the mucosal level. These can be influenced by inflammation and, in turn, modulate local immune responses, intestinal sensory function, and gut barrier integrity^76,86^. EGCs modulate these processes after injury by responding to and/or secreting inflammatory mediators such as interleukin (IL)-1 and IL-6^87^. In response to pro-inflammatory stimuli released during inflammatory conditions (e.g. Crohn’s disease, chagasic megacolon) EGCs express major histocompatibility group class II antigens to activate a specific subset of lymphocyte (regulatory T [Treg] cell) to maintain homeostasis^88,89^. Interestingly, Rao *et al*. used the Plp1 promoter to selectively eliminate glia in mice and found that EGCs are not required for epithelial homeostasis but have a role to play in the regulation of intestinal motility in a sex-dependent manner^90^.

With regard to GI motility, the intestinal peristaltic reflex is also regulated by bidirectional communication between enteric neurons and EGCs by detecting neurotransmitter receptor signalling, particularly through excitatory neuronal pathways^86,91^. EGCs receive signals from enteric neurons via multiple neurotransmitters (e.g., acetylcholine, catecholamine, glutamate and serotonin)^86^ that activate intracellular calcium responses and evoke glial activities^69^. Reciprocally, glial activation leads to the release of gliotransmitters such as purinergic transmitters, ATP, through pannexin membrane channels and/or through the reversal of neurotransmitter transporters. This gliotransmission could modulate enteric neural circuits or be sufficient to drive intestinal neurogenic contractions^86,91,92^. Studies showed that augmentation or ablation of glial activation is capable of affecting the strength, frequency and velocity of colonic contractions and development of post-inflammatory visceral hypersensitivity^91,93,94^. EGCs have also been implicated in the development of post-operative ileus from the increased production of IL-1^95^.

In normal physiological conditions, EGCs work to maintain homeostasis within the ENS by consistently monitoring extracellular environments via their receptors. Alteration of ENS homeostasis, including through insults, evokes a process of reactive gliosis. This context-dependent process is aimed at the restoration of homeostasis and the limitation of tissue damage but can also contribute to disturbances of GI motility and visceral pain. In certain circumstances, reactive gliosis can become detrimental and lead to neuroinflammation and aberrant neuronal plasticity, eventually leading to dysfunctional abnormalities, including permanent dysfunction of EGCs (gliopathy)^76^. Much recent interest has been focused on the modulatory properties of EGCs for the treatment of different GI diseases, especially those characterised by chronic inflammation and altered neuroplasticity (e.g., disorders of GI hypersensitivity or motility). Most of these novel treatment strategies focus on preventing reactive gliosis^76^ (reviewed in 96).

## ENS interaction with the epithelium and sensory signalling

Overall, the ENS provides the critical sensory system that is required to function at the vast interface of the GI mucosal lining with the gut luminal contents, which contain both components essential for life and health (e.g., nutrients and symbionts) and those that are potentially injurious (pathogens and toxins). In turn, these sensory elements work in concert with effectors to evoke appropriate responses within the many component cells of the GI tract. Improved understanding in recent years has revealed far more complex and interactive functional roles for each of these interactions.

Projections from both enteric neurons and glia in the submucosal plexus directly contact the epithelial cells to suggest the role of the ENS in neuro-epithelial communication to control mucosal functions and maintain the intestinal epithelial integrity through cytokine production and regulation of tight junction expression^97–100^. The roles of glia in this respect have been discussed earlier. Recently, Jarret *et al*. found that enteric neurons play a key role in the mucosal barrier by releasing IL-18 to control goblet cell antimicrobial protein expression and to directly kill intestinal pathogens^101^.

Sensory signalling in the bowel is mediated via three basic cells: intrinsic primary afferent neurons (IPANs), enteroendocrine cells and immune cells^2^. IPANs form an interconnected network with their terminals encoding chemical stimuli from intermediate cells (e.g., enterochromaffin cells) located in the lamina propria of the gut as well as directly receiving mechanical stimuli from intestinal contractions and distensions. As the ENS is organised in microcircuits, sensory signals from IPANs are able to initiate local intramural reflexes via interneurons and enteric motor neurons^66,67^. This output is ultimately involved in the modulation of intestinal contraction, epithelial secretion and enteric blood flow^70^.

As the mucosal endings of sensory neurons are separated from intraluminal contents by epithelial lining, these contents are detected by receptors on enteroendocrine cells. These are specialised cells within the epithelium, which release messenger molecules, such as neurotransmitters, to activate both intrinsic and extrinsic sensory neurons and modulate ENS activity^102,103^. Enterochromaffin cells constitute one subtype of enteroendocrine cell and function as sensory transducers responding to intraluminal stimuli. The main transmitter released from enterochromaffin cells onto nerve terminals is serotonin, whose release is necessary for generating neural signals for normal peristalsis and secretomotor reflexes^66,104^.

An example of neuro-endocrine integration in response to luminal stimuli is the control of gastric acid secretion from parietal cells, which involves three main factors: gastrin, histamine and somatostatin. These hormones are released from subtypes of enteroendocrine cells—antral G-type enteroendocrine cells, enterochromaffin-like cells and gastric mucosal D cells, respectively—in response to changes in luminal contents. Parietal cells are also influenced by excitatory neurons located within the gastric ENS, and the release of gastrin is also generated by a transmitter released from enteric neurons^105^.

## ENS regulation of immune function

The GI tract presents one of the largest immune organs in the body. There is recent and accumulating evidence to suggest that the ENS and immune systems are inexorably linked from development through to the neuromodulation of intestinal immunity by the ENS.

### Intestinal macrophages

Intrinsic enteric neurons, especially those of the myenteric plexus, have been identified as gatekeepers of immune homeostasis in the GI tract. A particular focus of this interaction has been the muscularis macrophages (MM), a macrophage subtype that resides within the myenteric plexus and associates with both enteric neurons and ICCs^17^ (reviewed in 106,107). Given this collaboration, it is perhaps not surprising that MM appear to be involved in the regulation of smooth muscle contractility and therefore of GI motility. Much of this understanding has come from the study of post-operative ileus, a well-described clinical state characterised by prolonged inertia of intestinal motility following trauma, surgery or instrumentation. MM appear to be key in the pathogenesis of post-operative ileus given their production of mediators that impair smooth muscle contractility, such as inducible nitric oxide synthase^108–110^. It is now clear that enteric neurons are capable of direct interaction with MM as well as modulating their function^111–114^. This interaction is likely to underlie the ability of vagal nerve stimulation to reduce post-operative ileus and inflammation by activating STAT3 in MM without direct contact between the vagal nerve and MM^115^. It is the myenteric neurons that appear to act as intermediaries between vagal efferents and MM, further supported by their ability to release critical macrophage colony-stimulating factor and respond to factors produced by MM (e.g., bone morphogenic protein)^113^. Interestingly, in intestinal inflammation, another population of macrophages, intraganglionic macrophages, appear to mediate degradation of the blood-myenteric barrier, which allows inflammatory mediators to access the myenteric ganglia. This may underlie a potential mechanism for disorders of neuronal dysfunction^116^.

### Adaptive immune system

There is evidence that the enteric nervous and immune systems share gene regulation and signalling mechanisms related to development (reviewed in 117). Furthermore, enteric neuron projections can be found in close apposition to adaptive immune cells, such as within Peyer’s patches (lymphoid follicles in the mucosa)^118,119^ and close to Treg cells in the lamina propria^120^. Vulchanova *et al*. found that enteric neurons were able to affect the balance of Treg cell populations in the colon^119^. The neurons release IL-6 to modulate immunoregulatory tone by inhibiting or activating different subtypes of Treg cells dependent on the IL-6 concentration (low concentrations decrease total *in vitro* Treg cells and increase the fraction of Treg cells that express the nuclear hormone receptor (ROR^+^), but high concentrations block both)^120^. The expression of IL-6 from enteric neurons can also be modulated by neuropeptide-releasing neurons (e.g., VIP^+^ neurons)^120^. The precise implications of these interactions for disease are yet to be determined.

## Conclusions and clinical implications

Though one of the most primordial systems of the human body, the GI tract remains arguably underrated. Yet it houses a ‘brain’, the ENS, whose interactions and functions truly span the breadth of physiological processes known to occur within the human body. This article has attempted to explore the most recent advances in our understanding of the role of the ENS in some of these. Beyond the basic development of the ENS, the last decade has witnessed a greater understanding of the diversity and complexity of the neurons and glia that compose the ENS and their elaborate interactions, not only with the enteric neuromusculature but also with epithelial, enteroendocrine and immune cells as well as microbiota and the CNS. The interactions, at minimum, effect and regulate luminal sensing, GI motility, epithelial function and integrity, immune function and blood flow and, beyond this, development and well-being across a number of organ systems. It follows that, though not addressed in this short review, disturbances of these roles and interactions of the ENS, especially in early life, are likely to have significant implications for the development of diseases of the GI tract, brain and other body systems. These include a host of conditions from disorders of gut-brain interaction (e.g., irritable bowel syndrome and functional dyspepsia) to severe GI motility (e.g., gastroparesis, paediatric intestinal pseudo-obstruction, slow transit constipation and Hirschsprung disease) and inflammatory disorders (e.g., necrotising enterocolitis and inflammatory bowel disease) as well as those affecting the CNS. These are covered in excellent reviews^52,121–125^. Therefore, a deeper understanding of the ENS and the molecular mechanisms of its roles and interactions appears essential in the drive to develop novel and effective preventative or therapeutic strategies for these often devastating diseases^126^.
